# Uranium rhodium bonding in heterometallic complexes†
†Electronic supplementary information (ESI) available. CCDC 1519919–1519921. For ESI and crystallographic data in CIF or other electronic format see DOI: 10.1039/c6dt04570g



**DOI:** 10.1039/c6dt04570g

**Published:** 2017-02-03

**Authors:** J. A. Hlina, J. A. L. Wells, J. R. Pankhurst, Jason B. Love, P. L. Arnold

**Affiliations:** a EaStCHEM School of Chemistry , University of Edinburgh , Joseph Black Building , The King's Buildings , Edinburgh EH9 3FJ , UK . Email: polly.arnold@ed.ac.uk

## Abstract

The heterotetra- and bimetallic uranium(iv)–rhodium(i) complexes [U^IV^I_2_(μ-OAr^P^-1κ^1^ *O*,2κ^1^ *P*)_2_Rh^I^(μ-I)]_2_ (**2**) (Ar^P^O^–^ = 2-(diphenylphosphino)-6-*tert*-butyl-4-methylphenoxide) and U^IV^I(μ-I)(μ-OAr^P^-1κ^1^ *O*,2κ^1^ *P*)_3_Rh^I^ (**3**) were prepared by treatment of U^IV^I(OAr^P^-κ^2^ *O*,*P*)_3_ (**1**) with rhodium(i) iodide olefin complexes. The reaction of **1** with the monodentate cyclooctene (coe) rhodium(i) precursor [(coe)_2_Rh^I^I]_2_ gives only the bimetallic complex [U^IV^Rh^I^] **3**, and with the diene [(cod)Rh^I^I]_2_ (**5**) (cod = 1,5-cyclooctadiene), mixtures of [U^IV^Rh^I^]_2_ complex **2** and [U^IV^Rh^I^] **3** along with (cod)Rh^I^OAr^P^-κ^2^ *O*,*P* (**4**), a Rh^I^ side-product from the formation of **2**. The complexes were characterised by single crystal X-ray diffraction, NMR and UV-vis-NIR spectroscopy, and electrochemistry. The U^IV^–Rh^I^ intermetallic distances in **2** (2.7601(5) Å) and **3** (2.7630(5) Å) are among the shortest between f-elements and transition metals reported to date. Despite almost identical U–Rh bond lengths in the solid state, in solution only weak, and very different interactions between the metal centres are found.

## Abstract 2

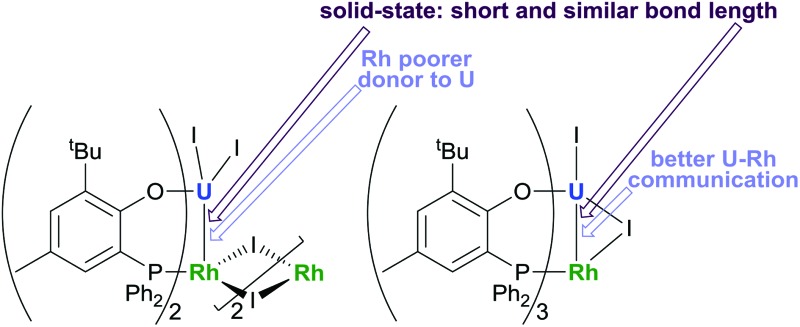
The U^IV^–Rh^I^ intermetallic distances in the U_2_Rh_2_ complex (left, 2.7601(5) Å) and URh complex (right, 2.7630(5) Å) are very short and almost identical in the solid state even though solution electrochemistry suggests very different metal-based reduction processes.

## Introduction

The chemistry of multimetallic transition metal complexes has received considerable interest in recent decades for its potential in catalytic applications and small molecule activation.^2,3^ The proximity of contrasting d-block metal centres can promote reactivity beyond the scope of homometallic compounds. An understanding of the interaction between the metal centres is key to enabling predictions of their reactivity and thus the development of multimetallic systems suitable for catalytic applications.

While many successes have resulted from a focus on the d-block metals, the manipulation of interactions between f-element cations and transition metal centres is an area that remains poorly understood. Heterobimetallic bonds with f-block elements are extremely rare, and homometallic bonds as yet unseen, in sharp contrast to much of d-block chemistry.^1^ The few reports of compounds that feature bonds between d- and f-block elements have begun to help to improve our understanding of the nature of metal–metal bonding. Furthermore, the 5f orbitals have sufficient spatial extension that renders the d–f intermetallic bond a possibility, and thus the isolation of actinide-transition metal complexes a particularly interesting target.

Complexes with bonds between uranium and transition metals are rare and limited to iron,^4–6^ ruthenium,^4,7^ cobalt,^8–10^ rhenium,^11–13^ group 10 metals,^14^ and silver.^15^ The first examples, Cp_3_U-MCp(CO)_2_ (M = Fe, Ru), were reported by Sternal and Marks in 1987.^4^ Although no crystallographic data was provided, analyses conclusively indicated metal–metal bonding rather than isocarbonyl bridging between the metal centres. This was based on their earlier work on thorium, which allowed the crystallographic verification of a thorium–ruthenium bond in Cp_3_Th-RuCp(CO)_2_.^16^ More recent examples of thorium-transition metal complexes include combinations with cobalt^10^ and copper.^17^

Complexes featuring unsupported d–f intermetallic bonds provide a ‘pure’ metal–metal interaction and are thus crucial for understanding the bonding but are inherently limited to d-block fragments possessing at least a partial negative charge, and systematic variations of fragments that provide a deeper bonding understanding are usually not possible for these isolated examples. More robust and diverse bonding situations, and reactivity can be explored by using supporting ligands that bridge the metal centres. The groups of Bart and Thomas demonstrated the use of bridging heterobidentate PN ligands to generate uranium(iv)–cobalt(i) complexes featuring short metal–metal bonds.^9^ Recently we showed that a bridging diphenylphosphine-substituted aryloxide ligand allows straightforward incorporation of neutral group 10 metal centres into a uranium(iv) complex, [U^IV^X(μ-OAr^P^-1κ^1^ *O*,2κ^1^ *P*)_3_M] (X = F, I, OSiMe_3_, M = Ni, Pd, Pt) enabling a comprehensive study of the metal–metal interactions, showing bond order was highest for the less polar U–Ni bond, and decreased going down the group, that replacing the uranium-bound iodide *trans* to the nickel centre with the more electronegative fluoride or siloxide also results in a strengthening of the U–Ni bond, and showing that U employs both its 5f and 6d orbitals in covalent bonding to a significant extent.^14^ Here we report the extension of this approach to metal–metal bonded uranium(iv)–rhodium(i) complexes.

## Results and discussion

### Synthesis

**Scheme 1:**
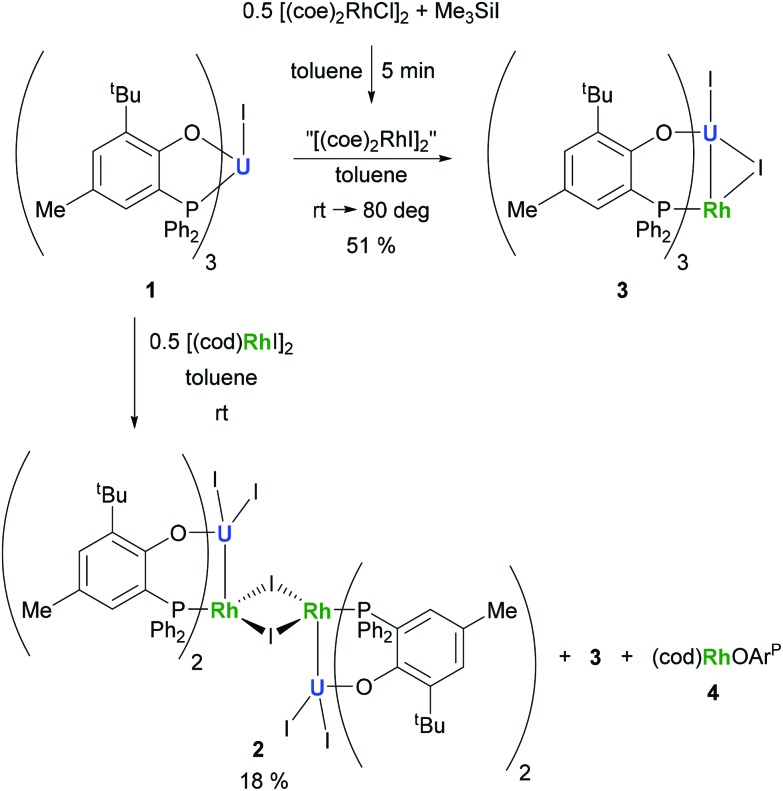
Reactions of **1** with [(cod)RhI]_2_ and *in situ* generated [(coe)_2_RhI]_2_, respectively.

**Fig. 1:**
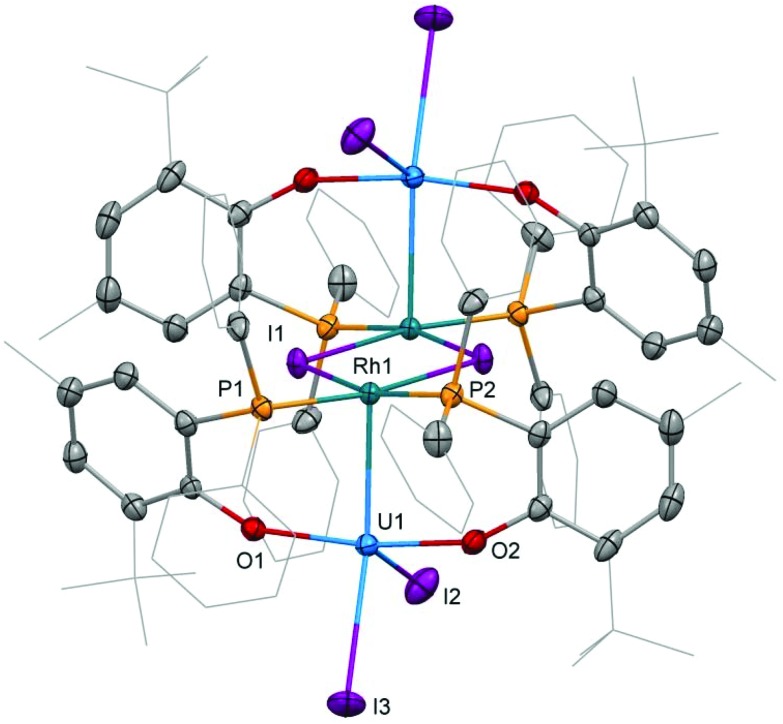
Molecular structure of **2**. Solvent molecules and hydrogen atoms are omitted, and peripheral carbon atoms are depicted as wireframe, for clarity. Thermal ellipsoids drawn at 50% probability. Selected distances (Å) and angles (°): U1–Rh1: 2.7601(5), U1–I2: 2.9559(5), U1–I3: 3.0239(5), U1–O1: 2.123(2), U1–O2: 2.131(2), Rh1–I1: 2.6902(6), Rh1–P1: 2.2830(9), Rh1–P2: 2.2936(8), O1–U1–O2: 164.01(8), I2–U1–I3: 92.87(1), P1–Rh1–P2: 99.86(3), I1–Rh1–I1′: 78.62(1).

The preparation of heterobimetallic uranium–rhodium complexes was envisioned *via* exchange of labile rhodium-bonded olefin ligands by the phosphine substituents of the uranium-bound aryloxide ligand, as we found this to be a convenient synthetic protocol to prepare bimetallic uranium-group 10 metal compounds.^14^ For this purpose, uranium(iv) tris[2-*tert*-butyl-4-methyl-6-(diphenylphosphino)phenolate] iodide, UI(OAr^P^)_3_ **1** (OAr^P–^ = 2-(diphenylphosphino)-6-*tert*-butyl-4-methylphenoxide), was treated with 1,5-cyclooctadienylrhodium(i) iodide, [(cod)RhI]_2_ **5** (cod = 1,5-cyclooctadiene), in toluene and the mixture allowed to stand at ambient temperature (Scheme 1). After 18 h dark green single crystals suitable for X-ray crystallography had formed, which were characterised as the tetrametallic uranium(iv)–rhodium(i) complex, [I_2_U(OAr^P^)_2_RhI]_2_ **2** (Fig. 1). As anticipated, the alkene ligands were readily replaced by the phosphine moieties of the uranium(iv) aryloxide to place rhodium in proximity to the uranium centre. But additionally, one phosphinoaryloxide ligand has been displaced from U by an iodide, with concomitant dimerisation of the resulting complex *via* bridging iodides, that saturates the coordination sphere of the rhodium centre. The NMR spectroscopic analysis of the supernatant shows the presence of two other products with ^31^P–^103^Rh coupling that give mass balance, paramagnetic U^IV^I(μ-I)(OAr^P^)_3_Rh^I^ (**3**) and diamagnetic (cod)Rh(OAr^P^) (**4**), Scheme 1. Attempts to separate the mixture were unsuccessful.

**Scheme 2:**
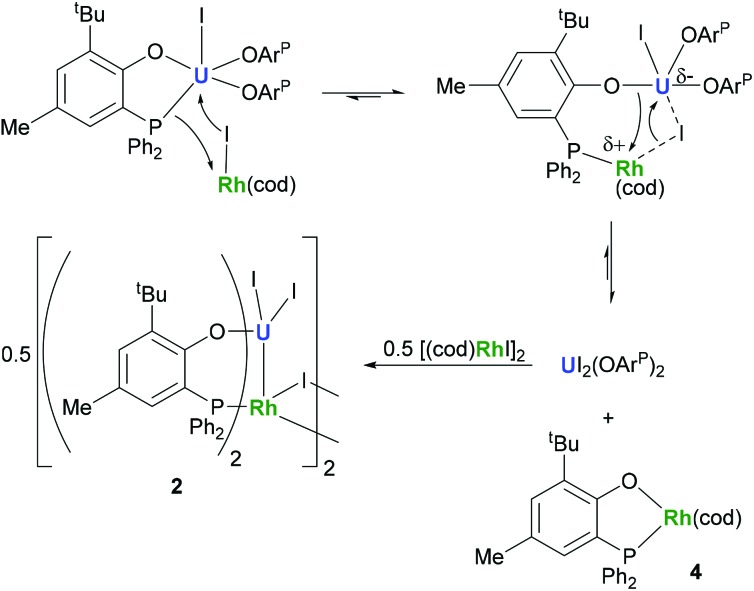
Proposed mechanism for the formation of **2** and **4**.

**Scheme 3:**
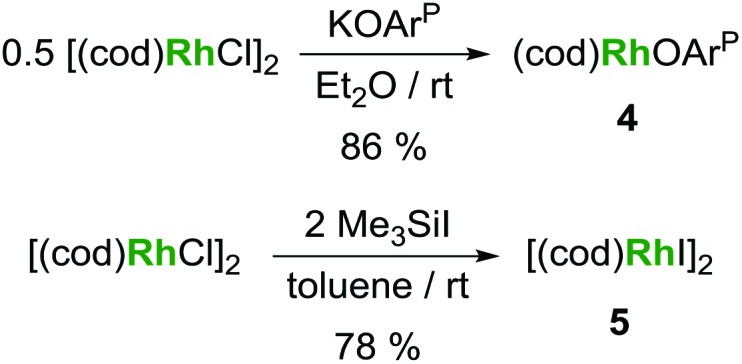
Alternative syntheses for (cod)Rh(OAr^P^) (**4**) and [(cod)RhI]_2_ (**5**).

The identity of the diamagnetic product as (cod)RhOAr^P^, **4**, is confirmed by comparison with an authentic sample prepared *via* reaction between KOAr^P^ and 0.5 equiv. of [(cod)RhCl]_2_ (Scheme 3) and supports the mechanism for the formation of **2** as suggested in Scheme 2. We presume that the synthesis of the dimeric compound **2** is related to a competing reaction pathway to alkene displacement resulting in the abstraction of the aryloxide from uranium. The coordination of the rhodium bound iodide to uranium would form an intermediary *ate*-complex that we suggest facilitates U–O bond fission to give monometallic compound **4**. The resulting UI_2_(OAr^P^)_2_ can react with another half equivalent of [(cod)RhI]_2_, yielding **2** upon dimerisation. This reactivity is somewhat similar to that of the bimetallic uranium(iv)–nickel(0) complex IU^IV^(OAr^P^)_3_Ni^0^ which upon oxidation forms Ni^II^(OAr^P^)_2_ as a result of aryloxide displacement from U.^14^

**Fig. 2:**
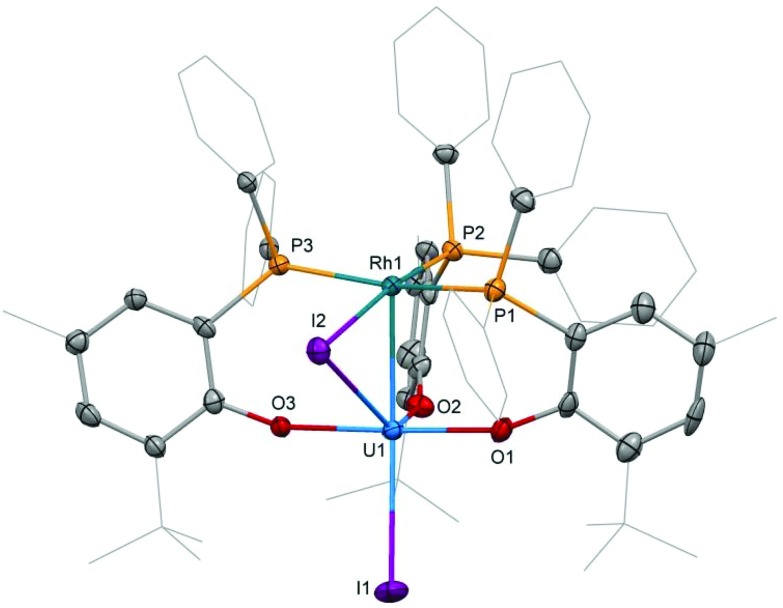
Molecular structure of **3**. Solvent molecules and hydrogen atoms are omitted and peripheral carbon atoms are depicted as wireframe for clarity. Thermal ellipsoids drawn at 50% probability. Selected distances (Å) and angles (°): U1–Rh1: 2.7630(5), U1–I1: 3.0428(5), U1–I2: 3.2264(5), U–O: 2.129(4)–2.156(3), Rh1–I2: 2.7296(6), Rh–P: 2.318(1)–2.416(1), I1–U1–Rh1: 167.75(2), U1–I2–Rh1: 54.51(1).

Based on the hypothesis that formation of the tetrametallic complex **2** is facilitated by a strongly binding olefin on the rhodium centre we investigated the use of monodentate cyclooctene (coe) to generate a more labile source of Rh. Translating the synthesis of [(cod)RhI]_2_ **5**, which was conveniently prepared by treatment of [(cod)RhCl]_2_ with trimethylsilyl iodide (Scheme 3), to the coe analogue [(coe)_2_RhI]_2_ did not give an isolable product. This is not surprising as [(coe)_2_RhCl]_2_ was reported to be significantly less stable than the corresponding cod derivative.^18^ Hence, this rhodium(i) source was prepared *in situ* by reaction of [(coe)_2_RhCl]_2_ with iodotrimethylsilane followed by treatment with **1** to give U^IV^I(μ-I)(OAr^P^)_3_Rh^I^ **3** (Scheme 1). Although the reaction readily proceeds at ambient temperature, the mixture was heated to 80 °C for 1 h to ensure the exchange of remaining uranium-bound chloride for iodide. After work-up **3** can be isolated as green crystals in 51% yield. Crystallographic analysis confirms the heterobimetallic nature of the dinuclear uranium(iv)–rhodium(i) complex **3** (Fig. 2).

A comparison with the data from the synthesis of the tetrametallic complex **2** shows that the inseparable paramagnetic side-product is indeed the bimetallic compound **3**. As both **2** and **3** are formed from [(cod)RhI]_2_ the rate determining steps in the respective synthetic pathways appear to have comparable rate constants.

### Crystallography

Single crystal X-ray diffraction of the two heterobimetallic uranium(iv)–rhodium(i) compounds showed intermetallic distances of 2.7601(5) (**2**) and 2.7630(5) Å (**3**) which are remarkably similar considering the differences in the coordination spheres of the metal centres (Fig. 1 and 2). These distances are among the shortest reported for uranium-transition metal complexes using ligands other than the bidentate OAr^P–^ ligand.^5–15^ However they are significantly longer than the intermetallic distances of our *o*-phosphinoaryloxide-supported uranium–palladium complex IU^IV^(OAr^P^)_3_Pd^0^ (U–Pd: 2.686(2)–2.694(1) Å).^14^

The U–O and U–I bond distances in **2** (U–O: 2.123(2) and 2.131(2) Å, U–I: 2.9559(5) and 3.0239(5) Å) and **3** (U–O: 2.129(4)–2.156(3) Å, U1–I1: 3.0428(5) Å) are within range of those observed for uranium-group 10 metal complexes featuring the same ligand.^14^ Only the bridging iodide in **3**, U1–I2: 3.2264(5) Å, is elongated in comparison with the terminally uranium-bound iodine atoms.

The rhodium ligand bond distances in **2** are Rh–I: 2.6902(6)/2.7169(2) Å and Rh–P: 2.2830(9)/2.2936(8) Å. The structural motif of halide-bridged rhodium complexes found in **2** is very common among rhodium(i) halide compounds, however only two crystal structures are reported for bis(triorganophosphine)rhodium(i) iodide dimers: [(dippp)RhI]_2_ (dippp = 1,3-bis(di-iso-propylphosphino)propane) and [Ac(xanthphos)RhI]_2_[BF_4_]_2_ (xanthphos = 9,9-dimethyl-4,5-bis(diphenylphosphino)xanthene).^19,20^ The molecular structure of the latter, featuring an acetyl group in the apical position on each rhodium centre, shows similar Rh–I, 2.698(1) and 2.703(1) Å, and Rh–P, 2.320(2) and 2.330(2) Å, distances.

In case of the dinuclear complex **3** Rh–I, 2.7296(6) Å, and Rh–P, 2.318(1)–2.416(1) Å, distances appear slightly longer than in **2**. A comparison with the molecular structure of (Ph_3_P)_3_RhI shows shorter Rh–P, 2.2303(6)–2.3239(8) Å distances.^21^ The terminal Rh–I bond, 2.6840(3) Å, is significantly shorter than the Rh–I bond in **3** as the latter is also bridging to the uranium centre.

The crystallographic analysis of monometallic rhodium(i) complex **4** shows a square planar arrangement of the ligand sphere (Fig. S1 in ESI†). The chelating Ar^P^O^–^ ligand Rh–O and Rh–P distances are 2.037(1) and 2.2676(5) Å, respectively, and a O–Rh–P bite angle of 83.36(4)°. The Rh–C distances to the cod ligand, 2.105(2)–2.216(2) Å, are similar to those found for other (cod)Rh^I^ complexes.

### Spectroscopy

In contrast to **3**, tetrametallic **2** is insoluble in solvents such as benzene or THF and only sparingly soluble in dichloromethane, in which both multimetallic compounds show significant decomposition within hours. The ^1^H NMR data of **2** in CD_2_Cl_2_ shows the phenolate-related shifts in a range of 26.78–53.40 ppm and the phenyl proton resonances at –12.84 to –8.83 ppm (see ESI Fig. S14 and S16†). The latter under-integrate, similarly to previous reports of uranium-group 10 metal compounds.^14^ As expected for **3** two sets of ^1^H resonances for the phenolate are observed in a 2 : 1 ratio at ranges of 18.93–41.97 ppm and –19.27––4.94 ppm, respectively. However, the phenyl resonances of the ligand are not observed which is probably due to signal broadening related to the influence of the paramagnetic uranium centre combined with a higher fluxionality when compared with the more rigid structure in **2**. The ^31^P{^1^H} NMR spectra of the two multimetallic compounds show strongly broadened doublets at 111.6 ppm (^1^ *J*_P–Rh_ = 145 Hz in CD_2_Cl_2_), **2**, and –218.5 ppm (^1^ *J*_P–Rh_ = 166 Hz in CD_2_Cl_2_)/–227.4 ppm (^1^ *J*_P–Rh_ = 160 Hz in C_6_D_6_), **3**. A second ^31^P NMR resonance, as expected from the ^1^H NMR data of **3**, could not located by an increase of data collection time and changes of the spectral window. The ^31^P–^103^Rh couplings are similar to what was observed for other triarylphosphine complexes of rhodium(i) including **4**, ^1^ *J*_P–Rh_ = 164 Hz (*δ* = 33.5 ppm in C_6_D_6_). The recording of ^103^Rh NMR was not studied as, in addition to the considerable efforts involved in ^103^Rh NMR spectroscopy, we were not able to observe resonances for heteronuclei (F, Si, Pt) in proximity of U(iv) in the related complexes.^14^

The electronic spectra of pyridine solutions of the complexes **2–4** are dominated by the absorptions of the aromatic ligand system with maxima ranging between 307 nm (*ε* = 9.5 × 10^3^ M^–1^ cm^–1^) in **4** to 312 nm (*ε* = 2.8 × 10^4^ M^–1^ cm^–1^) in **2**. In **4** this overlaps with the cod ligand π–π* transition.^22^ The spectra of the multimetallic compounds feature broad shoulders at around 510 nm (*ε* = 1.2 × 10^4^ M^–1^ cm^–1^), which is somewhat similar to what was observed for the heterobimetallic uranium-group 10 metal complexes.^14^ The monometallic rhodium compound **4** shows a second absorption maximum at 412 nm (*ε* = 2.3 × 10^3^ M^–1^ cm^–1^), relating to a metal to ligand charge transfer of the chelating phenolate ligand.^22^ In the NIR region the U–Rh complexes feature quite similar absorptions which can be assigned to U(iv) f–f transitions. However, unambiguous assignment of absorptions to metal-to-metal charge-transfer bands is not possible at this point

### Electrochemistry

We recently showed the monometallic IU(OAr^P^)_3_ complex undergoes a single irreversible reduction at *E*cp –2.87 V *versus* ferrocene, which was assigned to the U(iv)/U(iii) redox couple. Here, the electrochemistry of the Rh(i) complex **4** was investigated by cyclic voltammetry (CV), but in the electrochemical window provided by CH_2_Cl_2_/[^*n*^Bu_4_N][BPh_4_], complex **4** is completely redox-inactive.

In the cyclic voltammogram of the hetero-bimetallic complex **2**, only an irreversible reduction was observed, at the edge of the electrochemical window, at *E*cp –2.78 V. The CV of **2** is very similar to that of IU(OAr^P^)_3_, and indicates that the reduction process is localised on the U centre. This would suggest that, despite the short internuclear distance of 2.7601(5) Å, the Rh centre has virtually no electronic influence on the U centre, and thus the orbital interaction between the U(iv) and Rh(i) centres is very weak, at least in solution. Compared to IU(OAr^P^)_3_, there is a subtle change in the U(vi)/U(iii) reduction potential observed for **2**, which is attributed to a change in the coordination sphere around U. Other solution-phase behaviour of **2** also points to the dissociation of the dimeric structure at ambient temperature, but it is not clear whether this would have any effect on the U–Rh interaction. The evidence includes its dissolution then reaction with dichloromethane at room temperature and insolubility in boiling THF. We also note that there are numerous examples of room-temperature-active Rh(i) compounds of comparable structure whose catalytic activity is initiated by a dissociation step.

**Fig. 3:**
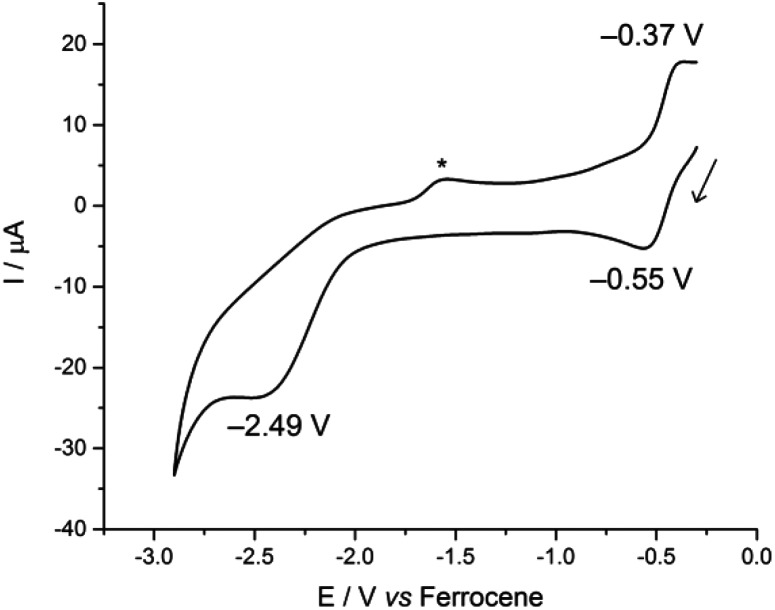
Cyclic voltammogram of **3**, measured at 100 mV s^–1^ in 0.1 M CH_2_Cl_2_/[^*n*^Bu_4_N][BPh_4_]. The asterisk denotes a decomposition product that is formed only after the irreversible reduction.

In contrast, the CV of complex **3** is more reminiscent of our previously reported U(iv)-group 10 heterobimetallic XU^IV^(OAr^P^)_3_M^0^ complexes. A *quasi*-reversible, one-electron oxidation process is observed at *E*ap –0.37 V, and an irreversible, two-electron reduction process is observed at *E*cp –2.49 V (Fig. 3). This concerted, multi-electron reduction process is extremely unlikely to be due to single-site reduction at U since actinide centres undergo one-electron redox processes. Instead, the two-electron reduction of **3** is most likely to be a two-electron occupation of a transition metal-based orbital, and is assigned as the Rh-based LUMO, in part, by analogy with the metal–metal anti-bonding molecular orbitals that we found to be the LUMO in the U(iv)-group 10 XU^IV^(OAr^P^)_3_M^0^ complexes.^14^ This assignment suggests that an orbital interaction between the U(iv) and Rh(i) centres exists only in complex **3**, which is surprising as the internuclear separation in that case is 2.7630(5) Å; longer than in **2** in the solid state. It may be that the frontier orbital is most readily delocalised across the two metals through the bridging iodide found only in **3**, and is retained in solution more readily, perhaps also because there are three (OAr^P^) bridging the U and Rh here. In comparison, **2** has no bridging iodide and only two OAr^P^ ligands; perhaps a weak interaction between the two metal centres is neither encouraged nor retained in solution.

## Conclusions

We have shown the preparation of heterobimetallic, multinuclear uranium(iv)–rhodium(i) complexes. The use of different olefin leaving groups on the rhodium precursor influences the product composition allowing access to different heterobimetallic uranium(iv)–rhodium(i) complexes. Very short, and very similar intermetallic distances are found in the solid state in these complexes, even though significantly shorter Rh–P distances in **2** imply that the Rh is a poorer donor to U in **2** than in **3**. In agreement, the electrochemical data indicate a weak interaction between the two metal centres in heterobimetallic **3**, although the frontier orbital being probed may well be delocalised across the two metals through the bridging iodide found only in **3**. In every case where electrochemistry suggests a U–M interaction so far (M = Ni, Pd, Pt, Rh in **3**) there are three (OAr^P^) ligands binding the two metals which effectively force the M to direct the filled d_*z*^2^_-orbital towards the U centre. The short U–Rh distance in the solid dimeric **2** may not even be retained in solution according to the electrochemical analyses.

In agreement with the finding from our analyses of the short but weak U–M bonds in the XU^IV^(OAr^P^)_3_M^0^ system,^14^ very short metal–metal distances do not necessarily confer strong metal–metal bonding.

## Experimental

### General details

All manipulations were carried out under a dry, oxygen-free dinitrogen atmosphere using standard Schlenk and glove box techniques. Benzene was distilled from potassium and stored over 4 Å molecular sieves. Hexane, THF, Et_2_O, and toluene were degassed and purified by passage through activated 4 Å molecular sieves or activated alumina towers and stored over 4 Å molecular sieves. Benzene-*d*_6_ was boiled over potassium, vacuum-transferred, and freeze–pump–thaw degassed prior to use. ^1^H, ^13^C, and ^31^P NMR spectra were recorded on Bruker AVA400, AVA500, AVA600, or PRO500 spectrometers at 300 K. Chemical shifts are reported in parts per million, *δ*, referenced to residual proton resonances, and calibrated against external TMS. Elemental analyses were carried out at London Metropolitan University, UK and Pascher Labor, Germany. IU(OAr^P^)_3_, [(cod)RhCl]_2_,^23^ [(coe)_2_RhCl]_2_ ^18^ were prepared according to published procedures. Trimethylsilyl iodide was stored over copper turnings and filtered immediately before use. All other reagents were purchased from standard suppliers and used as received. The UV-vis-NIR spectra were recorded on a JASCO V-670 spectrophotometer using a sealed quartz cuvette from solutions in pyridine.

Electrochemical measurements were carried out in 0.1 M solution of [^*n*^Bu_4_N][BPh_4_] in CH_2_Cl_2_, using an analyte concentration of 1 mM. The working electrode was a glassy-carbon disc, the counter electrode was a Pt gauze, and the quasi-reference electrode was a silver wire. All potentials were referenced against ferrocene.

Crystallographic data are deposited with the CCDC no. 1519919 (**4**), ; 1519920 (**2**), and ; 1519921 (**3**).

### U–Rh dimer (**2**)

IU(OAr^P^)_3_ (141 mg, 0.100 mmol, 2.0 eq.) and [(cod)RhI]_2_ (34 mg, 0.050 mmol, 1 eq.) were dissolved in toluene (4 ml) to give an orange solution which was allowed to stand for 18 h at ambient temperature during which time green crystals of **1** formed. The supernatant solution was separated and the crystalline product subsequently washed with toluene and hexane, respectively, and dried under reduced pressure. Dark green crystals of **1** (26 mg, 18%) were isolated. ^1^H NMR (*δ* in ppm, CD_2_Cl_2_, 300 K): –12.84 (s, 8H, Ph*H*), –10.21 (s, 4H, Ph*H*), –8.83 (br s, 8H, Ph*H*), 26.78 (s, 4H, Ar*H*), 29.43 (s, 12H, Ar*Me*), 50.60 (s, 36H, *tBu*), 53.40 (s, 4H, Ar*H*). ^31^P NMR (*δ* in ppm, CD_2_Cl_2_, 300 K): 111.5 (br d, ^1^ *J*_P–Rh_ = 145 Hz). Analysis calculated for C_92_H_96_I_6_O_4_P_4_Rh_2_U_2_: C 39.01. H 3.42. Found: C 40.08, H 3.77.

### U–Rh monomer (**3**)

IU(OAr^P^)_3_ (282 mg, 0.200 mmol, 1 eq.), [(coe)_2_RhCl]_2_ (72 mg, 0.10 mmol, 0.5 eq.), and trimethylsilyl iodide (40 mg, 0.20 mmol, 2 eq.) were slurried in toluene (5 ml) to give dark orange mixture. After 18 h the mixture had become dark green and was heated to 80 °C for 1 h followed by evaporation of all volatiles *in vacuo*. The dark green residue was taken up in a minimal amount of THF and subjected to centrifugation and filtration. The dark green solution was layered with hexane and stored at –20 °C to afford green crystals of **3** (167 mg, 51%). ^1^H NMR (*δ* in ppm, C_6_D_6_, 300 K): –19.27 (s, 9H, *tBu*), –14.59 (s, 1H, Ar*H*), –6.09 (s, 3H, Ar*Me*), –4.94 (s, 1H, Ar*H*), 18.93 (s, 6H, 2 × Ar*Me*), 27.06 (s, 2H, 2 × Ar*H*), 33.80 (br s, 18H, 2 × *tBu*), 41.97 (s, 2H, 2 × Ar*H*). ^31^P NMR (*δ* in ppm, C_6_D_6_, 300 K): –227.4 (br d, ^1^ *J*_P–Rh_ = 160 Hz). Analysis calculated for C_69_H_72_I_2_O_3_P_3_RhU: C 50.63. H 4.43. Found: C 50.42, H 4.55.

### (cod)Rh(OAr^P^) (**4**)

KOAr^P^ (77 mg, 0.20 mmol, 1.0 eq.) and [(cod)RhCl]_2_ (49 mg, 0.10 mmol, 0.50 eq.) were suspended in toluene (4 ml). The orange mixture was stirred for 18 h at ambient temperature. The solvent was evaporated under reduced pressure and the resulting orange residue extracted with hexane. The solvent was evaporated from the extract and the orange residue crystallized from Et_2_O at ambient temperature under slow evaporation giving orange crystals of **3** (96 mg, 86%). ^1^H NMR (*δ* in ppm, C_6_D_6_, 300 K): 1.70–1.89 (m, 4H, CH*H* cod), 1.75 (s, 9H, *tBu*), 2.06–2.21 (m, 4H, C*H*H cod), 2.11 (s, 3H, *Me*), 3.52–3.58 (m, 2H, C*H* cod), 5.70–5.78 (m, 2H, C*H* cod), 6.86 (ddd, *J* = 10, 2, 1 Hz, 1H, Ar*H*), 6.96–7.04 (m, 6H, *Ph*), 7.24 (d, *J* = 2 Hz, 1H, Ar*H*), 7.58–7.66 (m, 4H, *Ph*). ^13^C NMR (*δ* in ppm, C_6_D_6_, 300 K): 20.8, 28.6 (d, *J* = 1 Hz), 30.1, 33.1 (d, *J* = 2 Hz), 35.7 (d, *J* = 2 Hz), 68.9 (d, *J* = 13 Hz), 105.3 (dd, *J* = 11, 8 Hz), 117.1 (d, *J* = 49 Hz), 124.0 (d, *J* = 8 Hz), 128.9 (d, *J* = 10 Hz), 129.9 (d, *J* = 2 Hz), 130.2, 131.8 (d, *J* = 2 Hz), 133.2 (d, *J* = 12 Hz), 133.9 (d, *J* = 41 Hz), 139.0 (d, *J* = 12 Hz), 176.3 (d, *J* = 23 Hz). ^31^P NMR (*δ* in ppm, C_6_D_6_, 300 K): 33.5 (d, ^1^ *J*_P–Rh_ = 164 Hz). Analysis calculated for C_31_H_36_OPRh: C 66.67. H 6.50. Found: C 66.48, H 6.04.

### [(cod)RhI]_2_ (**5**)

In a modification of a literature preparation,^24^ [(cod)RhCl]_2_ (99 mg, 0.20 mmol, 1.0 eq.) was dissolved in toluene (3 ml) and trimethylsilyl iodide (80 mg, 0.40 mmol, 2.0 eq.) added to the stirred solution. The colour immediately changed from orange to red followed by precipitation of a red solid. The red-brown solid was separated, washed with hexane, and dried *in vacuo* to give 106 mg (78%) of [(cod)RhI]_2_. Analysis in agreement with published data.^25^
