# Supplementary material for: Uranium rhodium bonding in heterometallic complexes
Source: Dalton Trans. 2017 Feb 3;46(17):5540–5. doi: 10.1039/c6dt04570g (PMC5436036; doi:10.1039/c6dt04570g)
Supplement: Supplementary file 1 [file DT-046-C6DT04570G-s001.pdf]

Electronic Supplementary Material (ESI) for Dalton Transactions.
This journal is © The Royal Society of Chemistry 2017

SI: Uranium rhodium bonding in heterometallic complexes

Johann A. Hlina,[a,b] Jordann A. L. Wells,[a] James R. Pankhurst,[a] Jason B. Love,[a] and Polly L. Arnold[,a]*

[a]EaStCHEM School of Chemistry, University of Edinburgh, Joseph Black Building, The King's Buildings, Edinburgh EH9 3FJ, U.K.

[b]Current address: Institute of Inorganic Chemistry, Graz University of Technology, Stremayrgasse 9, 8010 Graz, Austria

Table of contents

1. Molecular structure of 4 and crystallographic data....2
  Figure S1. Molecular structure of **4**....2
  Table S1. Crystallographic data. 3
2. Electrochemistry 4
  Figure S2. Cyclic voltammogram of **2**. 4
  Figure S3. Cyclic voltammogram of **2** focussing on the irreversible red. feature only....5
  Figure S4. Square-wave voltammogram of **2**....6
  Figure S5. Cyclic voltammogram of **3** 7
  Figure S6. Cyclic voltammogram of **3** focussing on the quasi-reversible ox. feature only. 8
  Figure S7. Cyclic voltammogram of **3** focussing on the irreversible red. feature only....9
  Figure S8. Cyclic voltammogram of **3** with limited scanning range....10
  Figure S9. Linear-sweep voltammogram of **3** 11
  Figure S10. Square-wave voltammogram of **3** 12
  Figure S11. Cyclic voltammogram of **4** 13
3. UV-vis NIR spectra....14
  Figure S12: UV-vis spectra of 2 - 4 in the range 225 - 800 nm 14
  Figure S13: NIR spectra of 2 - 4 over the wavelength range 800 - 2100 nm....14
4. NMR spectra of 2....15
  Figure S14: ^{1}H NMR spectrum of 2 in CD_2Cl_2 at 300K.. 15
  Figure S15: ^{31}P NMR spectrum of 2 in CD_2Cl_2 at 300K....15
  Figure S16: Stacked ^{1}H spectra of 2 in CD_2Cl_2 at 300K at different time intervals....16

1. Molecular structure of **4** and crystallographic data

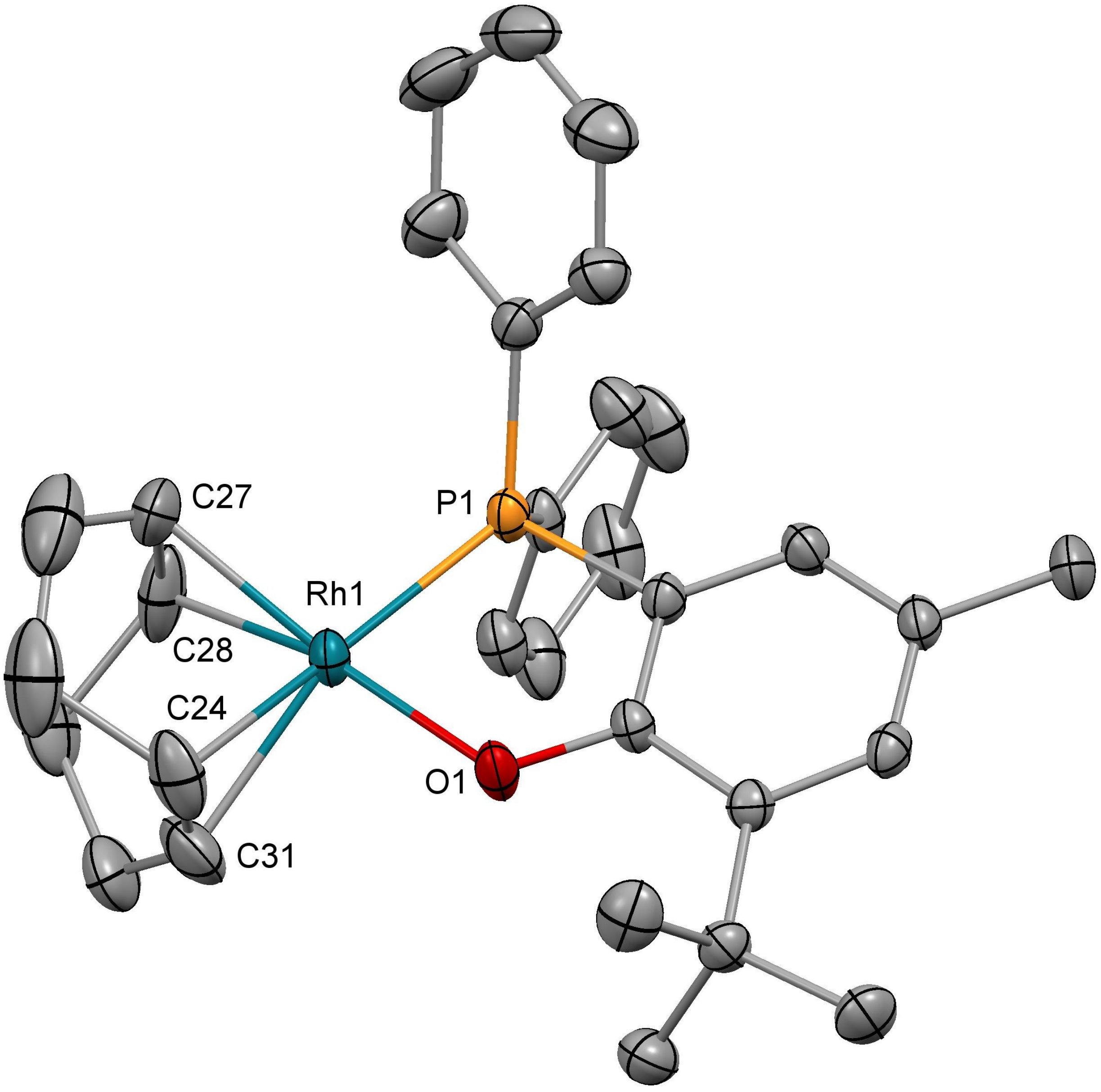

Figure S1. Molecular structure of **4**. Thermal ellipsoids drawn at 50 % probability and hydrogen atoms are omitted for clarity. Selected bond distances (Å) and angles (°): Rh1-P1: 2.2676(5), Rh1-O1: 2.037(1), Rh1-C24: 2.216(2), Rh1-C27: 2.105(2), Rh1-C28: 2.113(2), Rh1-C31: 2.198(2), P1-Rh1-O1: 83.36(4).

Table S1. Crystallographic data.

	**2**	**3**	**4**
CCDC number	1519920	1519921	1519919
Empirical formula	$C_{69}H_{72}I_2O_3P_3RhU$	$C_{92}H_{96}I_6O_4P_4Rh_2U_2$	$C_{31}H_{36}OPRh$
Formula weight	1636.91	2832.84	558.48
Temperature/K	170	170	170(2)
Crystal system	triclinic	monoclinic	Triclinic
Space group	P-1	$P2_1/n$	P-1
a/Å	12.3192(3)	17.06093(18)	9.7178(2)
b/Å	13.5642(3)	15.91912(19)	11.5025(2)
c/Å	26.1759(5)	19.8049(2)	12.6599(3)
α/°	98.8295(17)	90	102.7983(18)
β/°	97.8140(17)	97.6036(10)	102.8779(19)
γ/°	97.5610(17)	90	93.4883(18)
Volume/$Å^3$	4229.80(15)	5331.62(10)	1336.16(5)
Z	2	2	2
$\rho_{calc}g/cm^3$	1.285	1.765	1.388
μ/mm^{-1}	2.928	5.171	0.720
F(000)	1596.0	2664.0	580.0
Crystal size/mm^3	0.1842 × 0.1568 × 0.0464	0.5370 × 0.1936 × 0.1419	0.2734 × 0.2443 × 0.1046
2Θ range for data collection/°	6.062 to 54.968	5.656 to 54.966	6.122 to 54.97
Index ranges	$-15 \leq h \leq 15$, $-17 \leq k \leq 17$, $-33 \leq l \leq 33$	$-22 \leq h \leq 22$, $-20 \leq k \leq 20$, $-25 \leq l \leq 25$	$-12 \leq h \leq 12$, $-14 \leq k \leq 14$, $-16 \leq l \leq 16$
Reflections collected	97387	97286	30827
Independent reflections	19379 [R_{int} = 0.0881, R_{sigma} = 0.0803]	12211 [R_{int} = 0.0359, R_{sigma} = 0.0210]	6119 [R_{int} = 0.0280, R_{sigma} = 0.0225]
Data/restraints/parameters	19379/0/724	12211/0/504	6119/18/349
Goodness-of-fit on F^2	1.013	1.046	1.022
Final R indexes [I>=2σ (I)]	R_1 = 0.0442, wR_2 = 0.0859	R_1 = 0.0227, wR_2 = 0.0501	R_1 = 0.0246, wR_2 = 0.0615
Final R indexes [all data]	R_1 = 0.0715, wR_2 = 0.0929	R_1 = 0.0296, wR_2 = 0.0528	R_1 = 0.0279, wR_2 = 0.0630
Largest diff. peak/hole / e $Å^{-3}$	1.46/-1.02	0.93/-0.87	0.36/-0.31

2. Electrochemistry

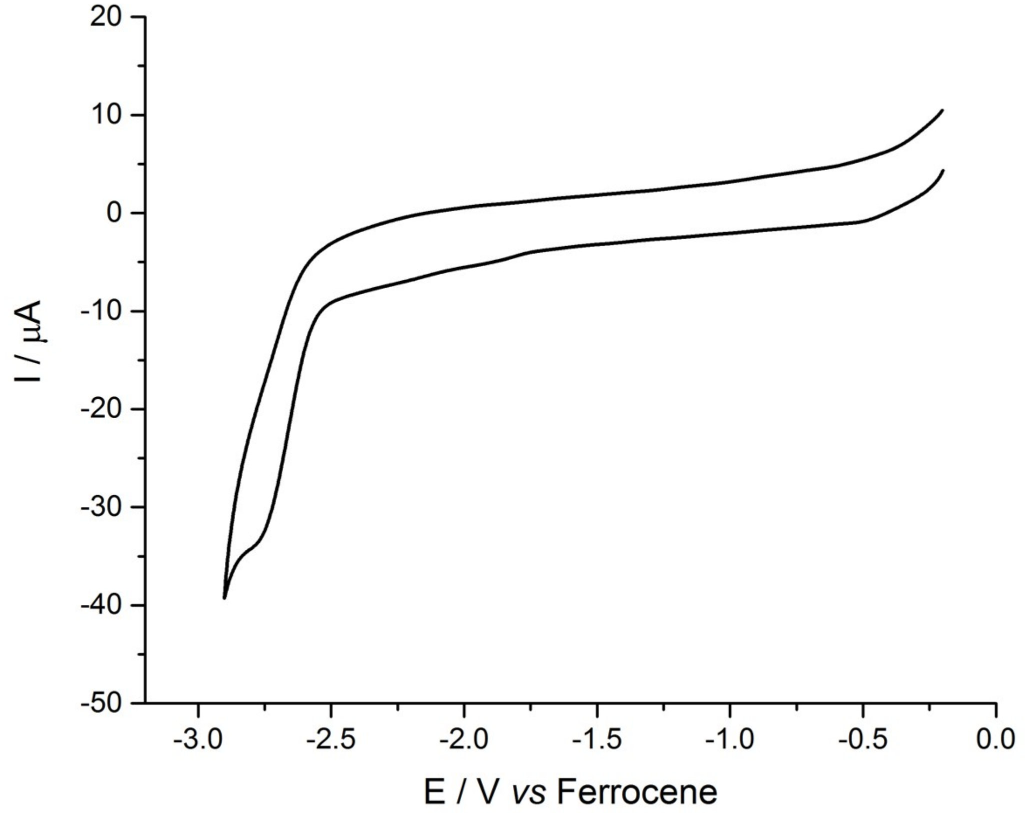

Figure S2. Cyclic voltammogram of **2**, measured at 100 mV s^{-1}, showing the full electrochemical window provided by CH_2Cl_2 / [$^{n}Bu_4$][BPh_4].

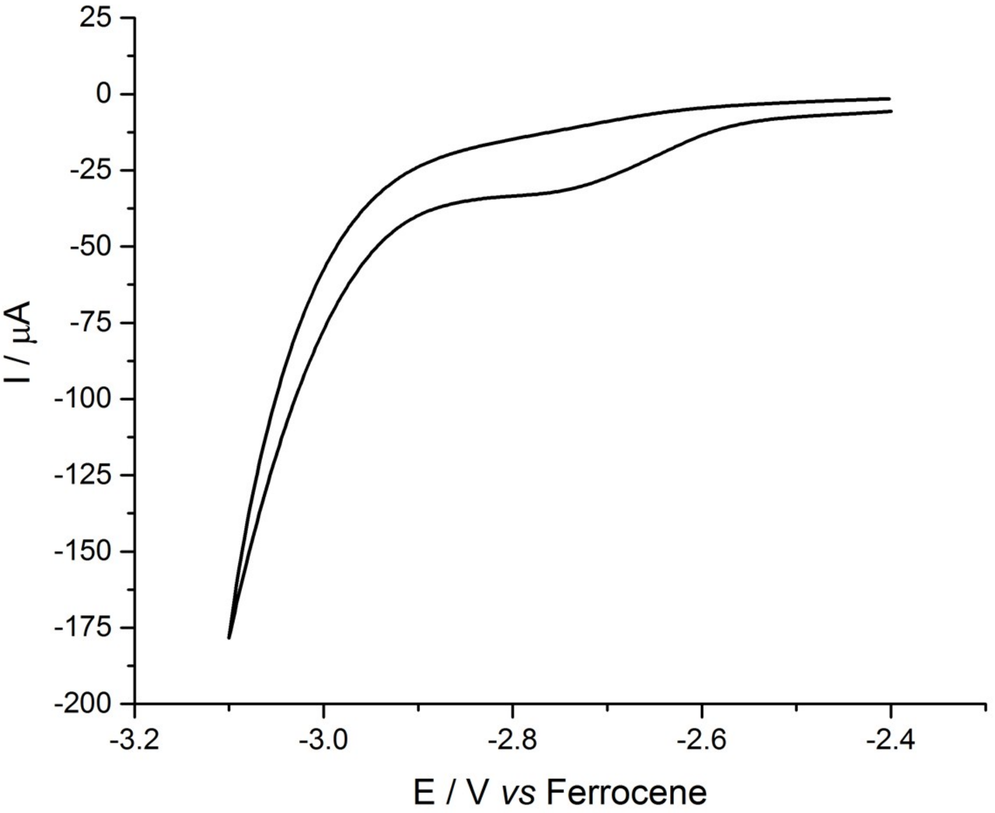

Figure S3. Cyclic voltammogram of **2**, measured at 100 mV s^{-1}, focussing on the irreversible reduction feature only.

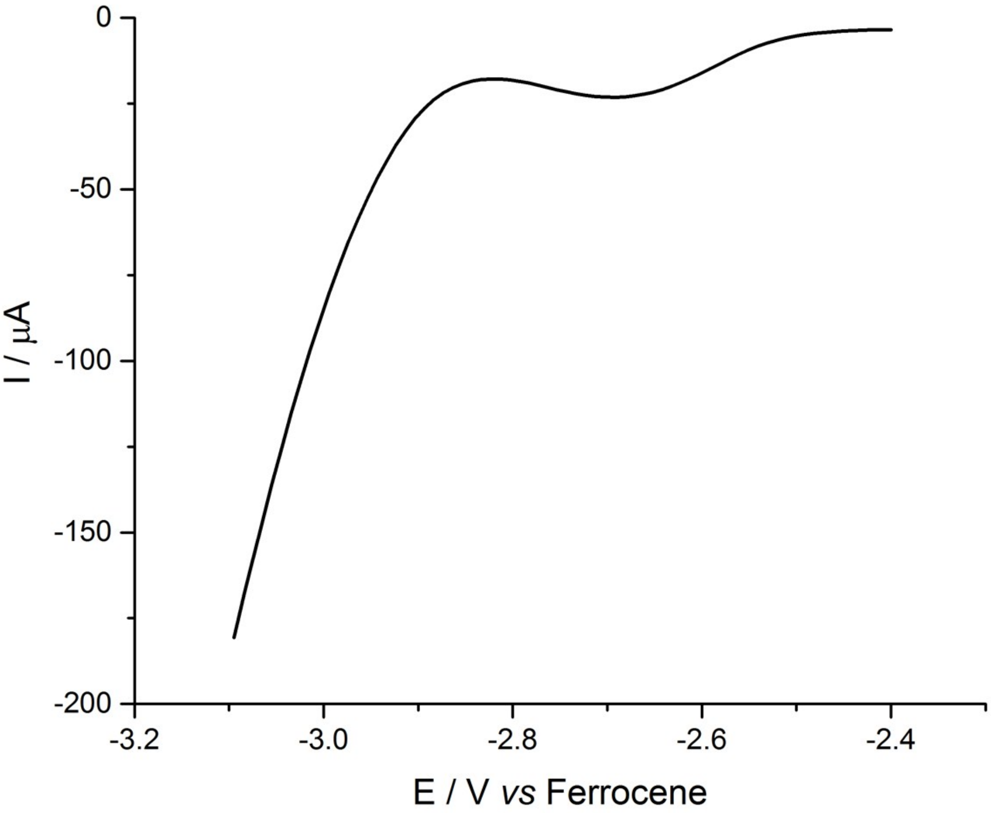

Figure S4. Square-wave voltammogram of **2**, showing only the cathodic scan. Frequency = 25 Hz; step potential = 5 mV; amplitude = 20 mV; scan rate = 124 mV s^{-1}.

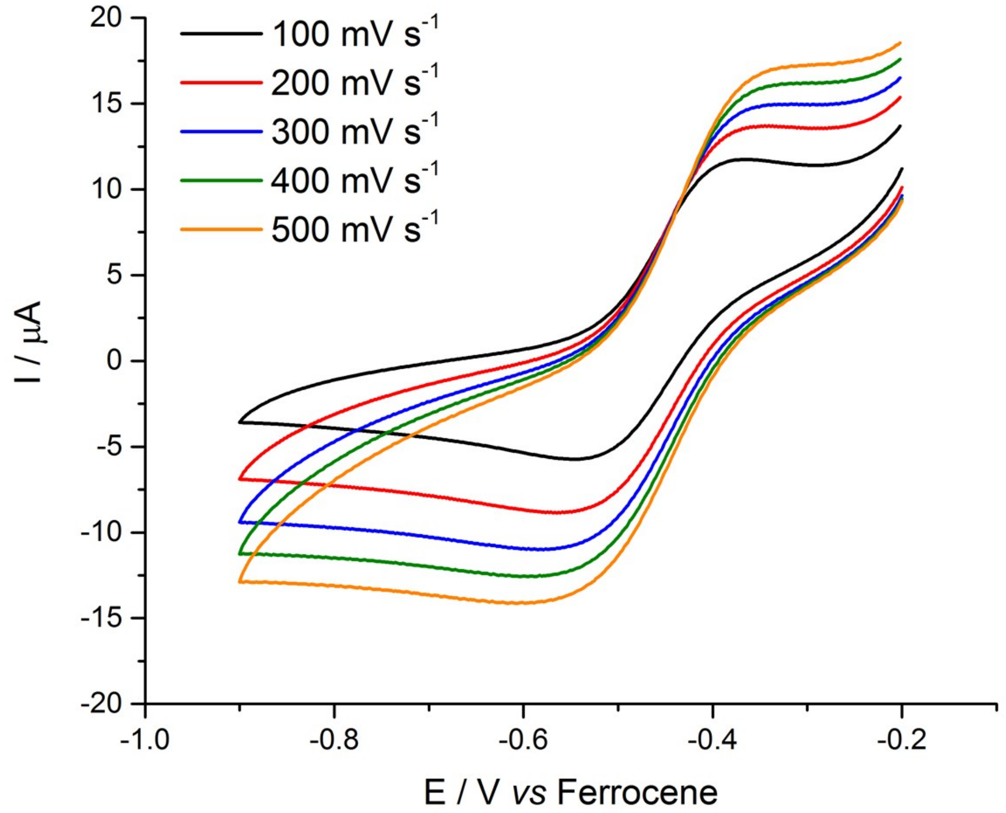

Figure S5. Cyclic voltammogram of **3**, focussing on the quasi-reversible oxidation feature only. Scan-rate dependence is observed for the peak potentials of both the oxidation and reduction waves.

It should be noted that DCM decomposes both bimetallic complexes **2** and **3**, but only after around 20 minutes. The voltammogram shown in Fig. 3 was recorded within 2 minutes of dissolution of **3** in the electrolyte. The data are reproducible and are considered sufficiently reliable for interpretation. We suggest the decomposition probably involves an oxidative addition of a Cl-C bond to Rh, which could be facilitated by initial U-coordination of DCM, generating a charged Rh(III) complex that can abstracts OAr^P from the U centre. Further work is in progress to test this theory.

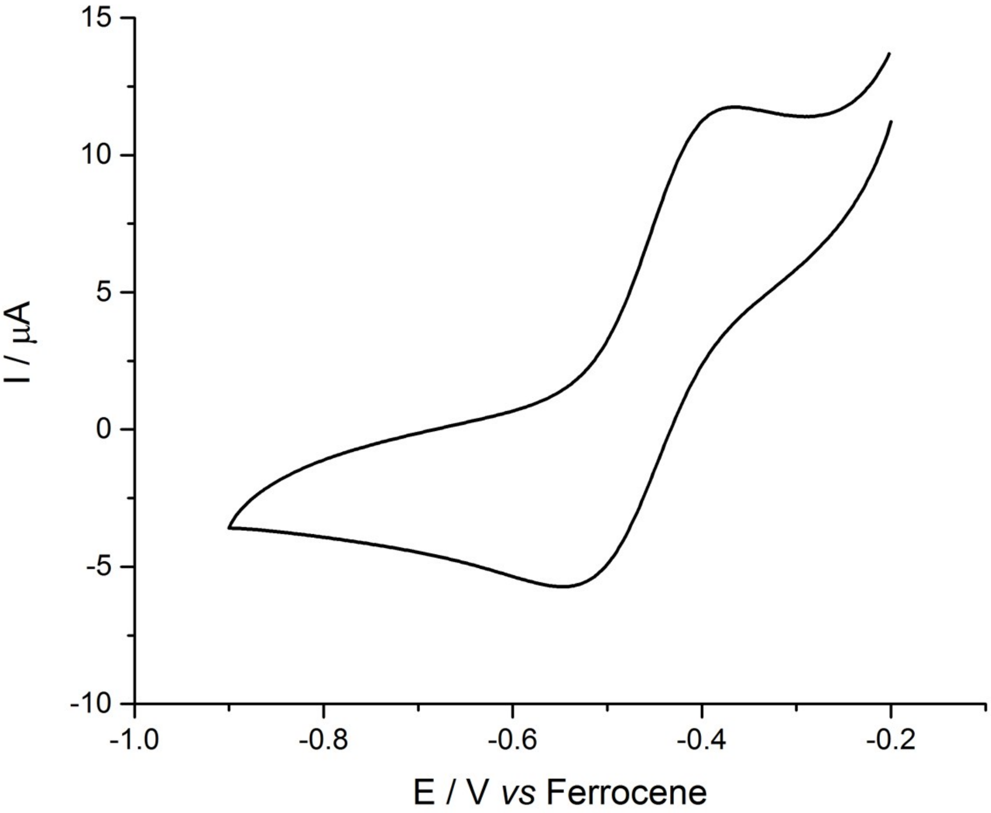

Figure S6. Cyclic voltammogram of **3**, measured at 100 mV s^{-1}, focussing on the quasi-reversible oxidation feature only.

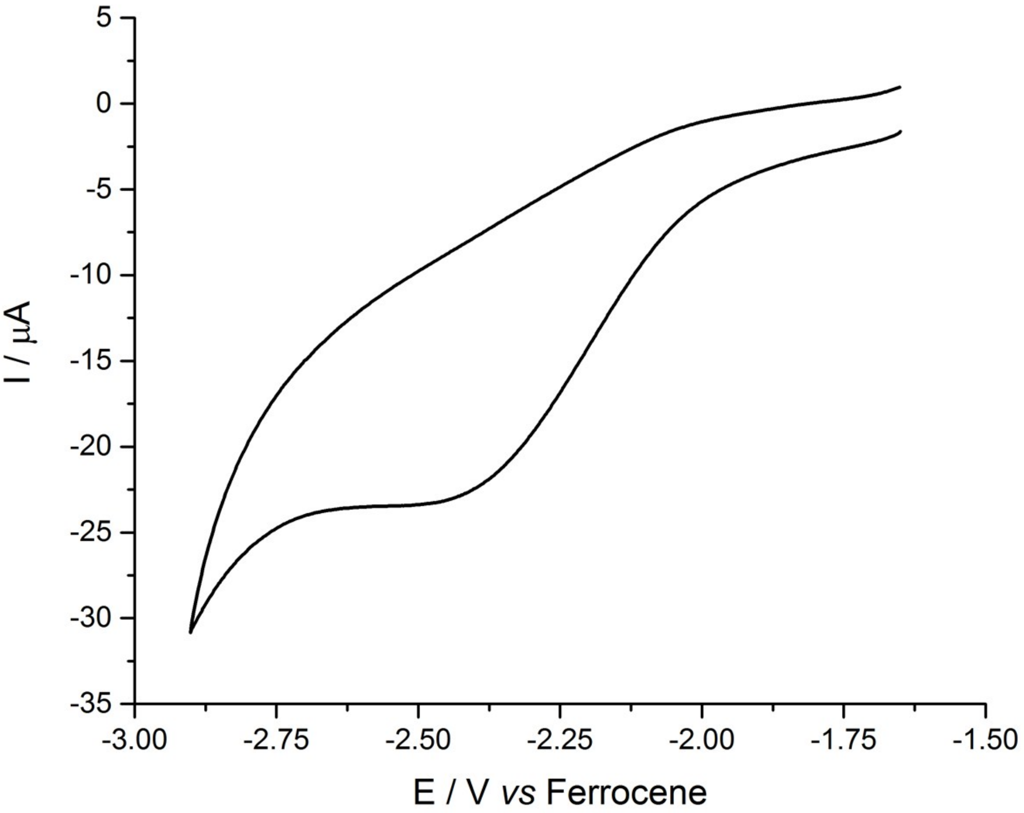

Figure S7. Cyclic voltammogram of **3**, measured at 100 mV s^{-1}, focussing on the irreversible reduction feature only.

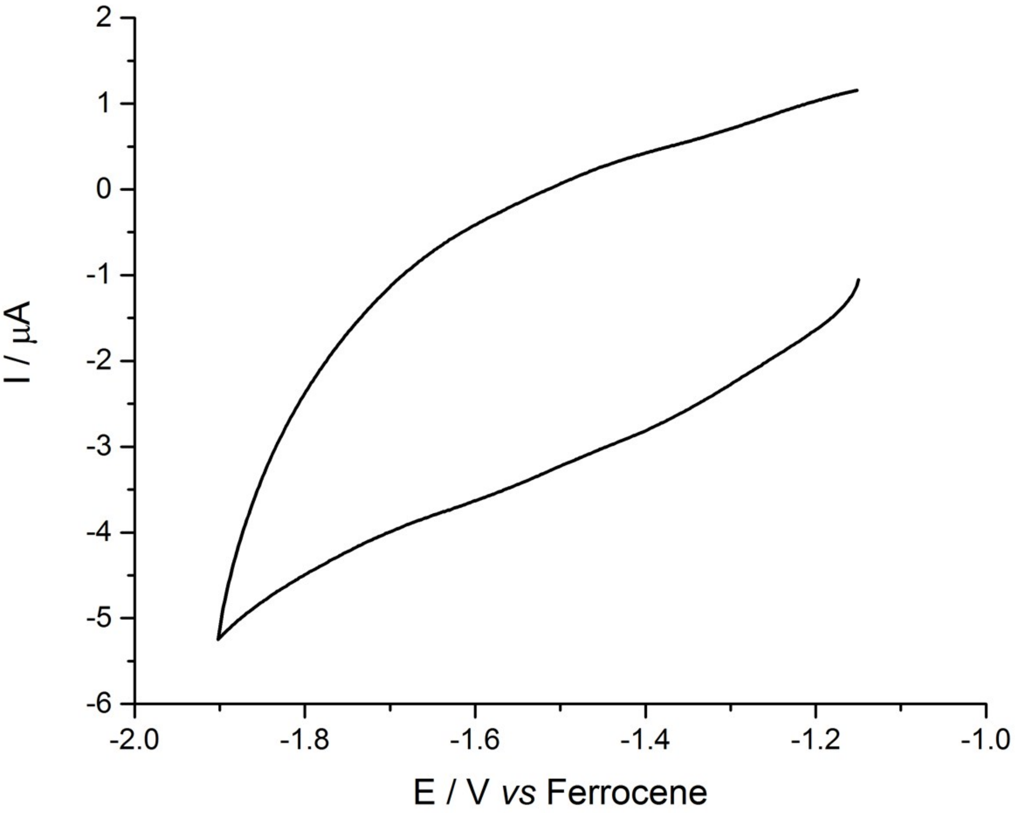

Figure S8. Cyclic voltammogram of **3**, measured at 100 mV s^{-1}, showing that the small oxidation feature that is observed at *ca*. -1.6 V in the full scan is not observed when scanning to only -1.9 V (shown here). The small feature in the full scan is therefore assigned to a decomposition product that is formed after scanning past -2.49 V.

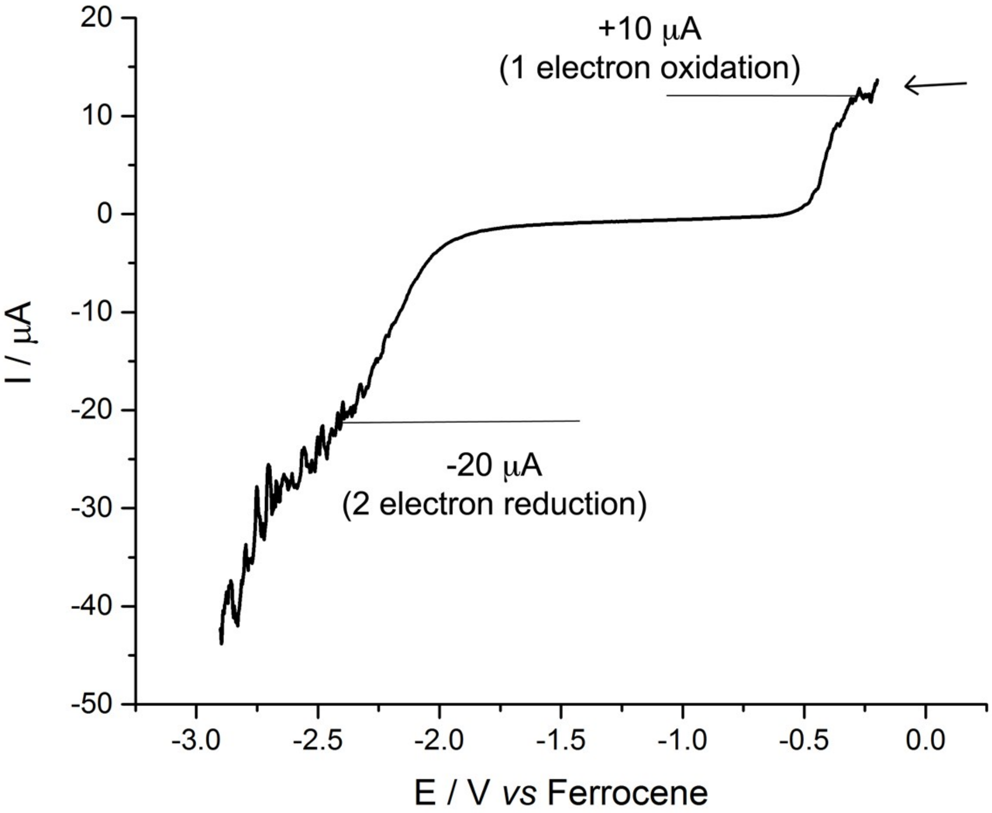

Figure S9. Linear-sweep voltammogram of **3**, measured at 5 mV s^{-1} as a stirred solution. The mass-transport limiting current for the oxidation process is *ca*. +10 μA, whereas that for the reduction process is ca. -20 μA. The number of electrons involved in the reduction is therefore twice that involved in the oxidation.

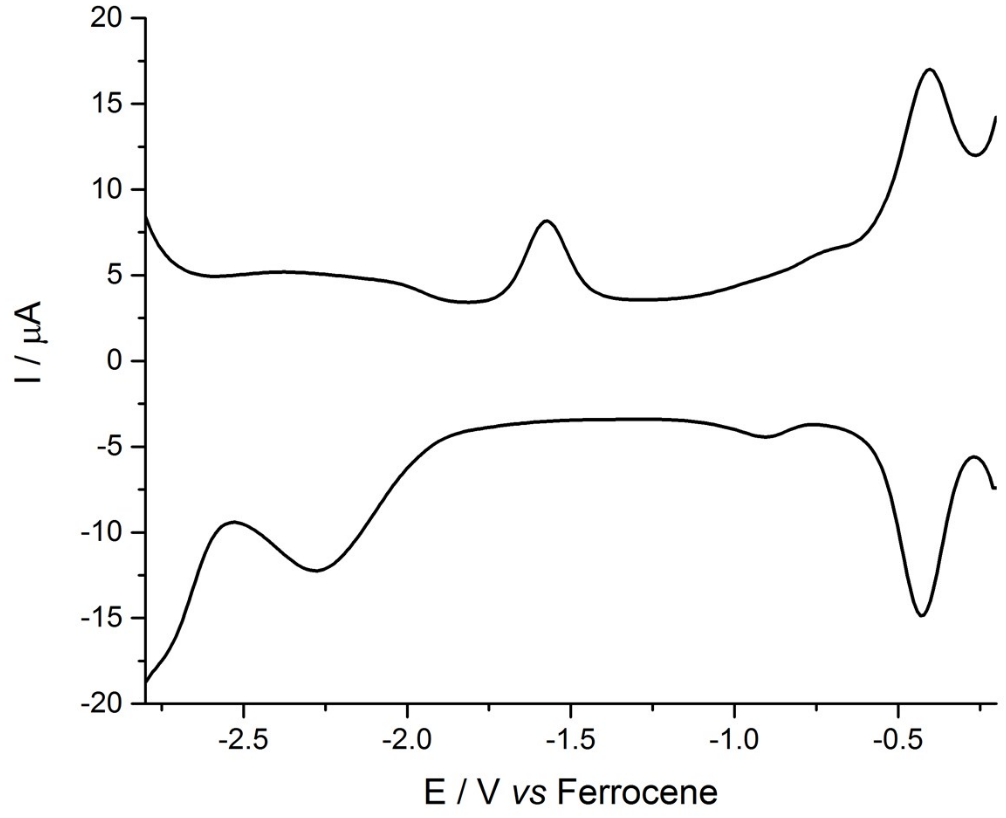

Figure S10. Square-wave voltammogram of **3**, showing forward and return scans measured for the full electrochemical window. Frequency = 25 Hz; step potential = 5 mV; amplitude = 20 mV; scan rate = 124 mV s^{-1}.

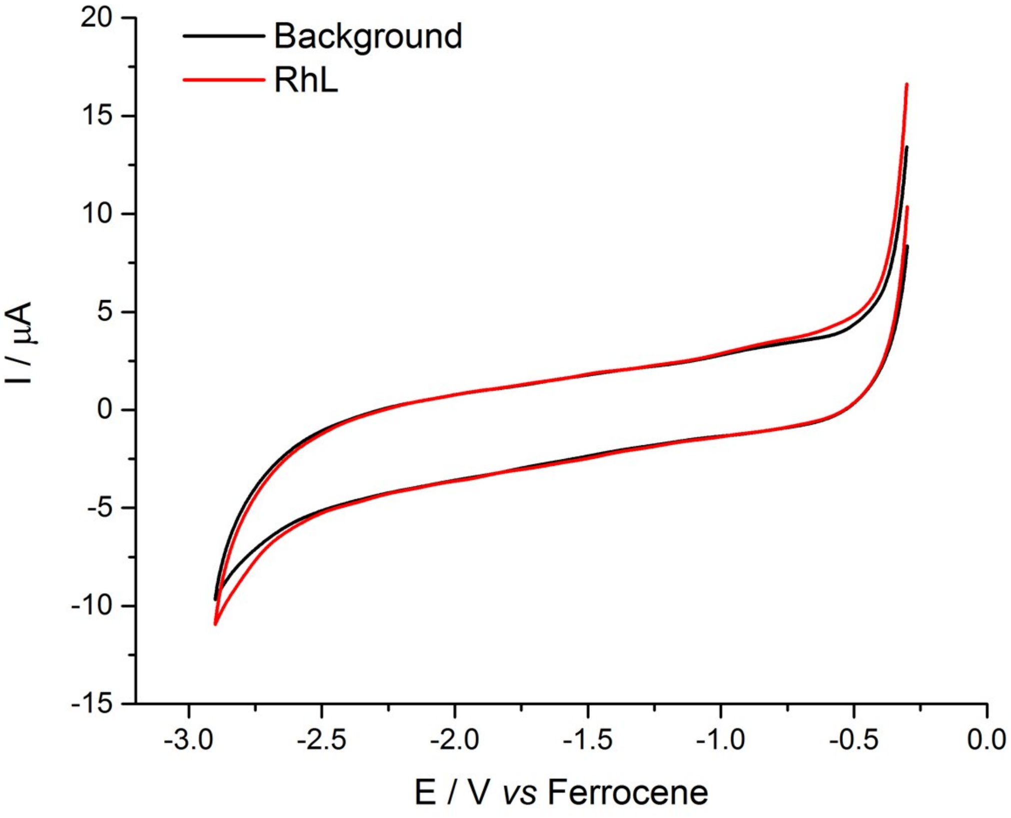

Figure S11. Cyclic voltammogram of **4**, measured at 100 mV s^{-1}. The background scan (measured before the compound was added to the solution) is overlaid in black to highlight the absence of any redox features in the electrochemical window.

3. UV-vis NIR spectra

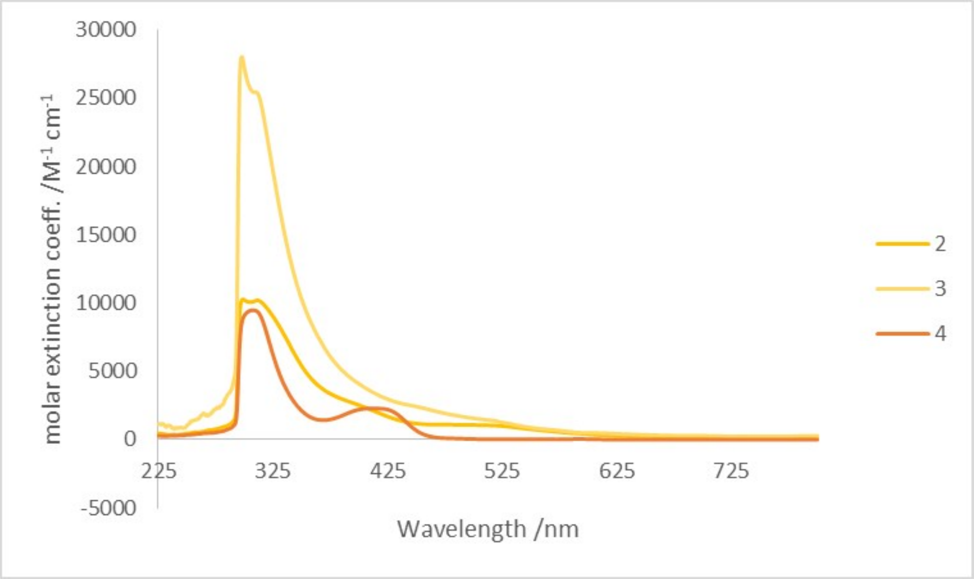

Figure S12: UV-vis spectra of **2** - **4** in the range 225 - 800 nm. Collected from 0.1 mM (**2**), 0.02 mM (**3**) and 0.1 mM (**4**) pyridine solutions respectively.

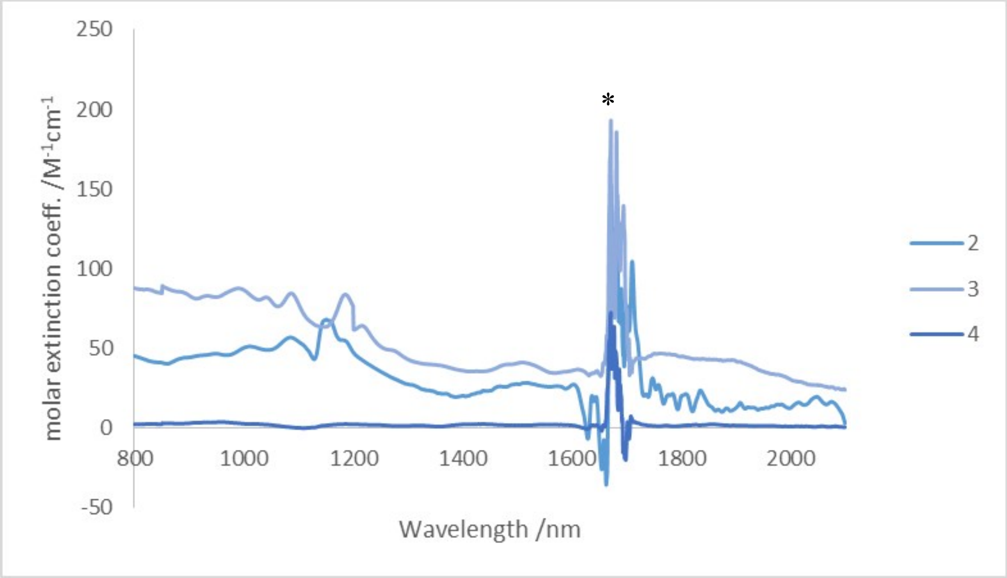

Figure S13: NIR spectra of **2** - **4** over the wavelength range 800 - 2100 nm. Spectra recorded from 1.0 mM (**2**), 0.4 mM (**3**) and 0.7 mM (**4**) solutions respectively. The asterisk marks solvent overtones.

4. NMR spectra of **2**

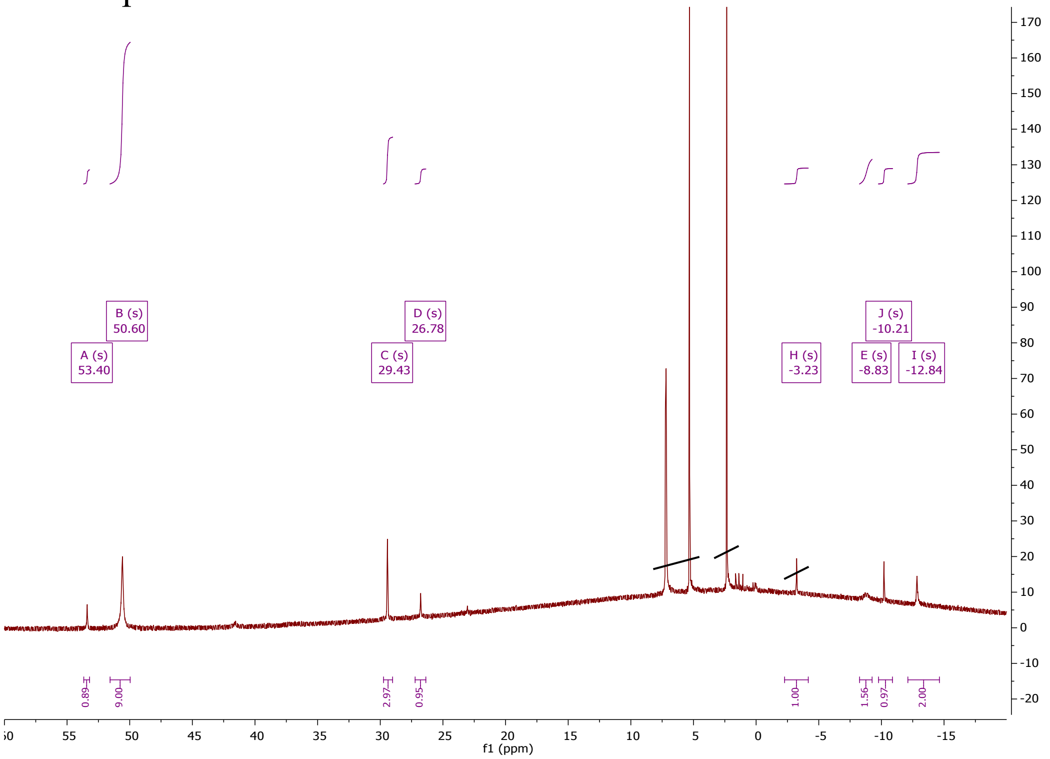

Figure S14: ^{1}H NMR spectrum of **2** in CD_2Cl_2 at 300K. Resonances corresponding to residual *proteo*-solvent and impurities are scored through.

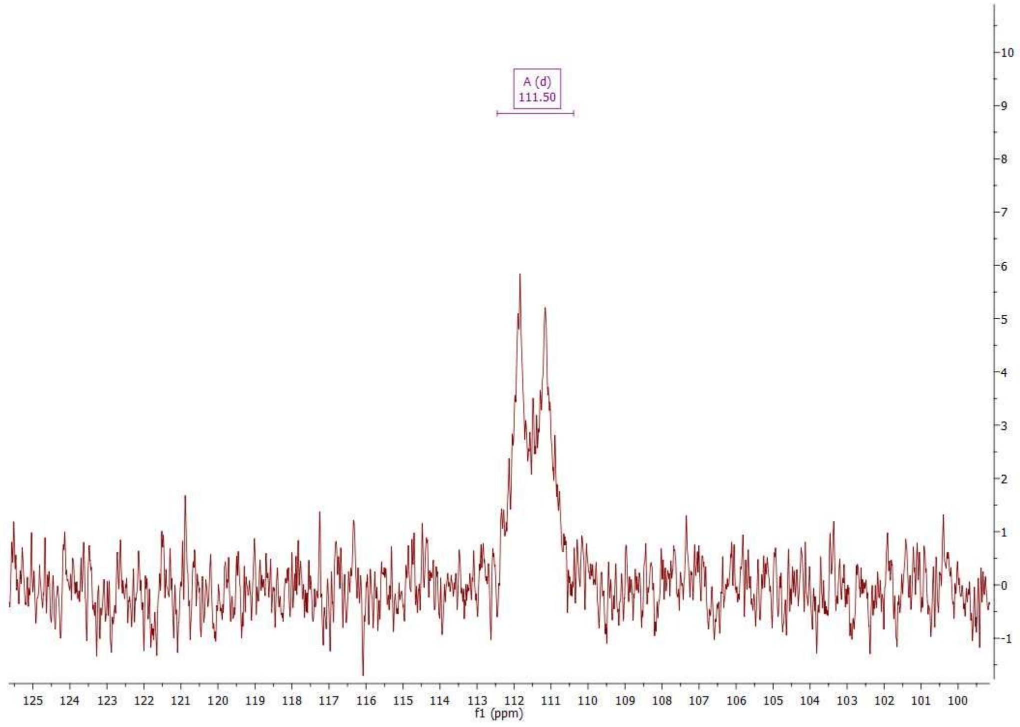

Figure S15: ^{31}P NMR spectrum of **2** in CD_2Cl_2 at 300K.

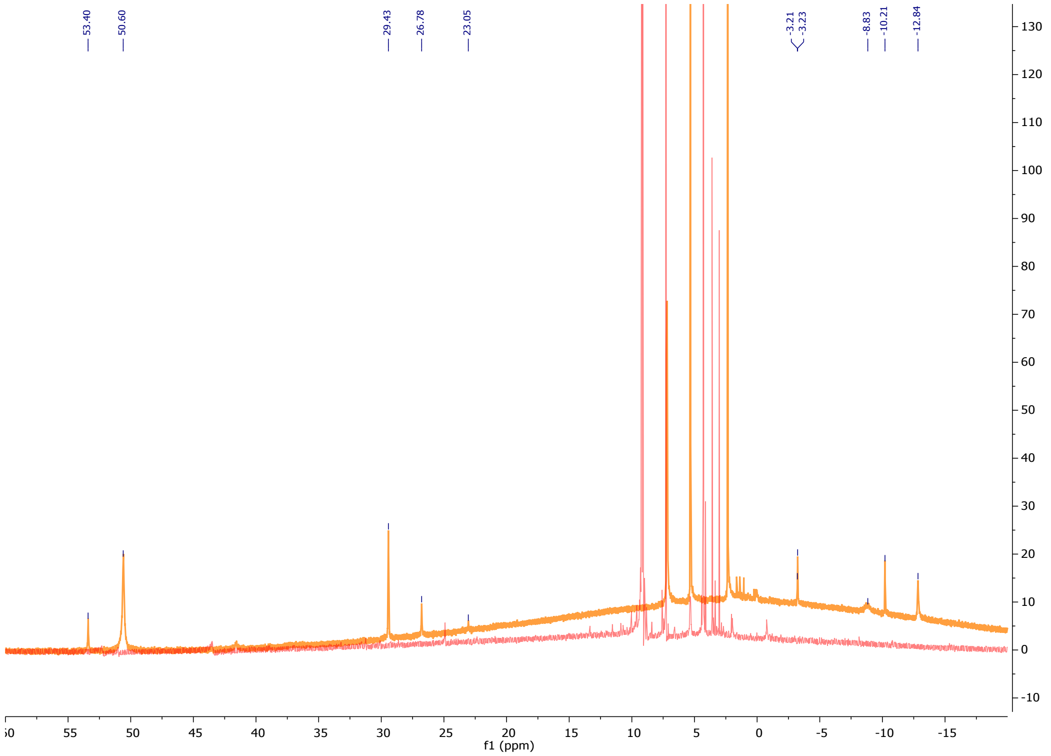

Figure S16: Stacked ^{1}H spectra of **2** in CD_2Cl_2 at 300K at different time intervals. The orange spectrum is t = 0 hours and the red spectrum is t = 72 hours.
